# Modeling platform to assess the effectiveness of single and integrated *Ixodes scapularis* tick control methods

**DOI:** 10.1186/s13071-024-06387-2

**Published:** 2024-08-12

**Authors:** Daniel Ruiz-Carrascal, Jonathan Bastard, Scott C. Williams, Maria Diuk-Wasser

**Affiliations:** 1 Department of Ecology, Evolution and Environmental Biology, Columbia University in the City of New York, New York, NY USA; 2 International Research Institute for Climate and Society, Columbia University in the City of New York, New York, NY USA; 3 Department of Environmental Science and Forestry, Center for Vector Biology & Zoonotic Diseases, The Connecticut Agricultural Experiment Station, New Haven, CT USA

**Keywords:** Lyme disease, Tick control, Interventions, Effectiveness, Simulation, Mathematical model

## Abstract

**Background:**

Lyme disease continues to expand in Canada and the USA and no single intervention is likely to curb the epidemic.

**Methods:**

We propose a platform to quantitatively assess the effectiveness of a subset of *Ixodes scapularis* tick management approaches. The platform allows us to assess the impact of different control treatments, conducted either individually (single interventions) or in combination (combined efforts), with varying timings and durations. Interventions include three low environmental toxicity measures in differing combinations, namely reductions in white-tailed deer (*Odocoileus virginianus*) populations, broadcast area-application of the entomopathogenic fungus *Metarhizium anisopliae*, and fipronil-based rodent-targeted bait boxes. To assess the impact of these control efforts, we calibrated a process-based mathematical model to data collected from residential properties in the town of Redding, southwestern Connecticut, where an integrated tick management program to reduce *I.xodes scapularis* nymphs was conducted from 2013 through 2016. We estimated parameters mechanistically for each of the three treatments, simulated multiple combinations and timings of interventions, and computed the resulting percent reduction of the nymphal peak and of the area under the phenology curve.

**Results:**

Simulation outputs suggest that the three-treatment combination and the bait boxes–deer reduction combination had the overall highest impacts on suppressing *I. scapularis* nymphs. All (single or combined) interventions were more efficacious when implemented for a higher number of years. When implemented for at least 4 years, most interventions (except the single application of the entomopathogenic fungus) were predicted to strongly reduce the nymphal peak compared with the no intervention scenario. Finally, we determined the optimal period to apply the entomopathogenic fungus in residential yards, depending on the number of applications.

**Conclusions:**

Computer simulation is a powerful tool to identify the optimal deployment of individual and combined tick management approaches, which can synergistically contribute to short-to-long-term, costeffective, and sustainable control of tick-borne diseases in integrated tick management (ITM) interventions.

**Graphical Abstract:**

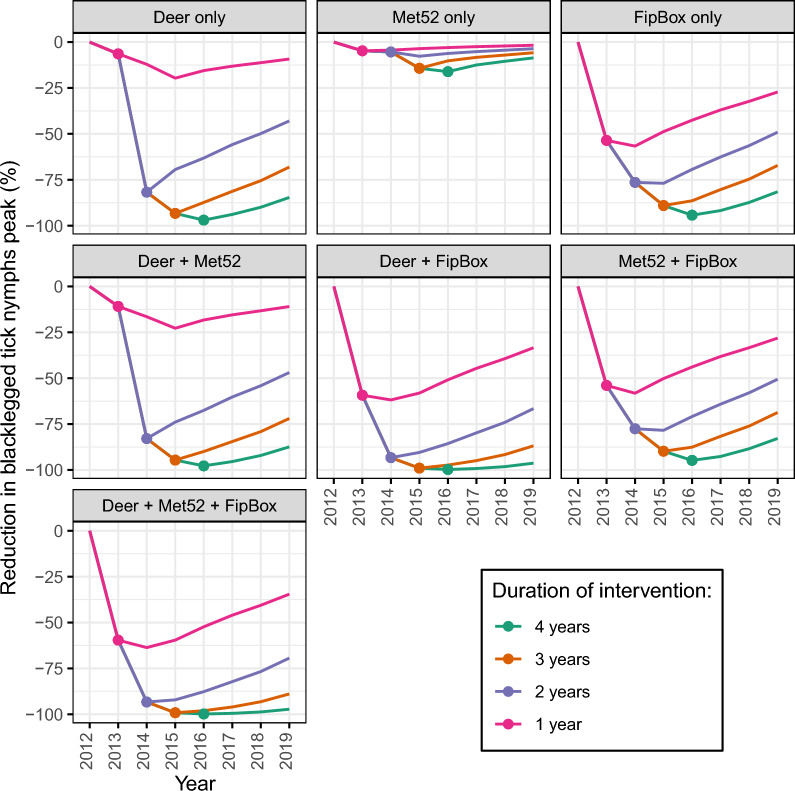

**Supplementary Information:**

The online version contains supplementary material available at 10.1186/s13071-024-06387-2.

## Background

Tick-borne diseases are expanding globally [1–3]. In Canada and the USA, the number of cases of one of these zoonotic diseases, Lyme disease, is on a steady rise [4–8]. This long-term trend has been attributed to the range expansion of the primary vector to humans, the blacklegged tick, *Ixodes scapularis* Say [9–11]. Complementary approaches have been developed to reduce human exposure to the causative agent of Lyme disease, *Borrelia burgdorferi*. One group of interventions targets reduction in density of ticks in the environment by environmental and host-targeted acaricides [12–18], another aims to decrease infection prevalence of hosts and ticks [15, 18, 19], and the other aims to reduce human-tick encounters [17, 20]. Here we focus on methods suppressing *I. scapularis* abundance in the environment, which include area-wide application of natural and synthetic acaricidal compounds, landscape and vegetative cover modifications and management [14], host-targeted synthetic acaricides such as fipronil-based rodent-targeted bait boxes [15, 16], biological control [17, 18, 20, 21] by entomopathogenic fungal agents [18, 20, 21], and reductions of white-tailed deer, *Odocoileus virginianus*, populations [22, 23].

Integrated tick management (ITM) has been proposed to be more effective than single measures [24, 25]. However, there is limited empirical support [12, 13, 26–28] and knowledge gaps on the effectiveness of such integrated tick control methods remain [20, 29]. A significant barrier in evaluation and optimization of ITM approaches is that intervention outcomes are often assessed as the reduction in density of nymphs/infected nymphs, but there is a lack of mechanistic understanding about what component of the enzootic cycle was impacted. For example, when assessing the effect of white-tailed deer management, the direct effect of deer reduction is often not assessed. Rather, the focus tends to be on deer reduction impacts to the entomological inoculation rate (measured as the density of questing  *I. scapularis* nymphs infected with the target pathogen), which is usually the metric of human health risk. While useful in assessing effectiveness of the intervention in reducing human risk in the particular context assessed, this black-box evaluation limits our ability to expand the application to other settings or optimizing different arms of an ITM approach.

Here we present a robust, empirically calibrated modeling platform that can simulate and compare single and combined interventions, including differences in timing of delivery. Mathematical modeling approaches provide a mechanistic understanding of the tick–host system. They also represent the system’s continuous (i.e., not restricted to specific periods) dynamics in simulations, and thus capture the temporal complexity of intervention implementation. Using an adaptation of an extensively validated climate-driven model [30, 31], we propose a modeling platform to evaluate the impact of individual and multiple tick management approaches on the density of questing *I. scapularis* nymphs (DON), a key component of Lyme disease risk [32]. Hereafter we refer to *I. scapularis* as ‘tick’ and specific *I. scapularis* stages as ‘larvae’, ‘nymphs’ and ‘adults.’

## Methods

### General approach

**Fig. 1:**
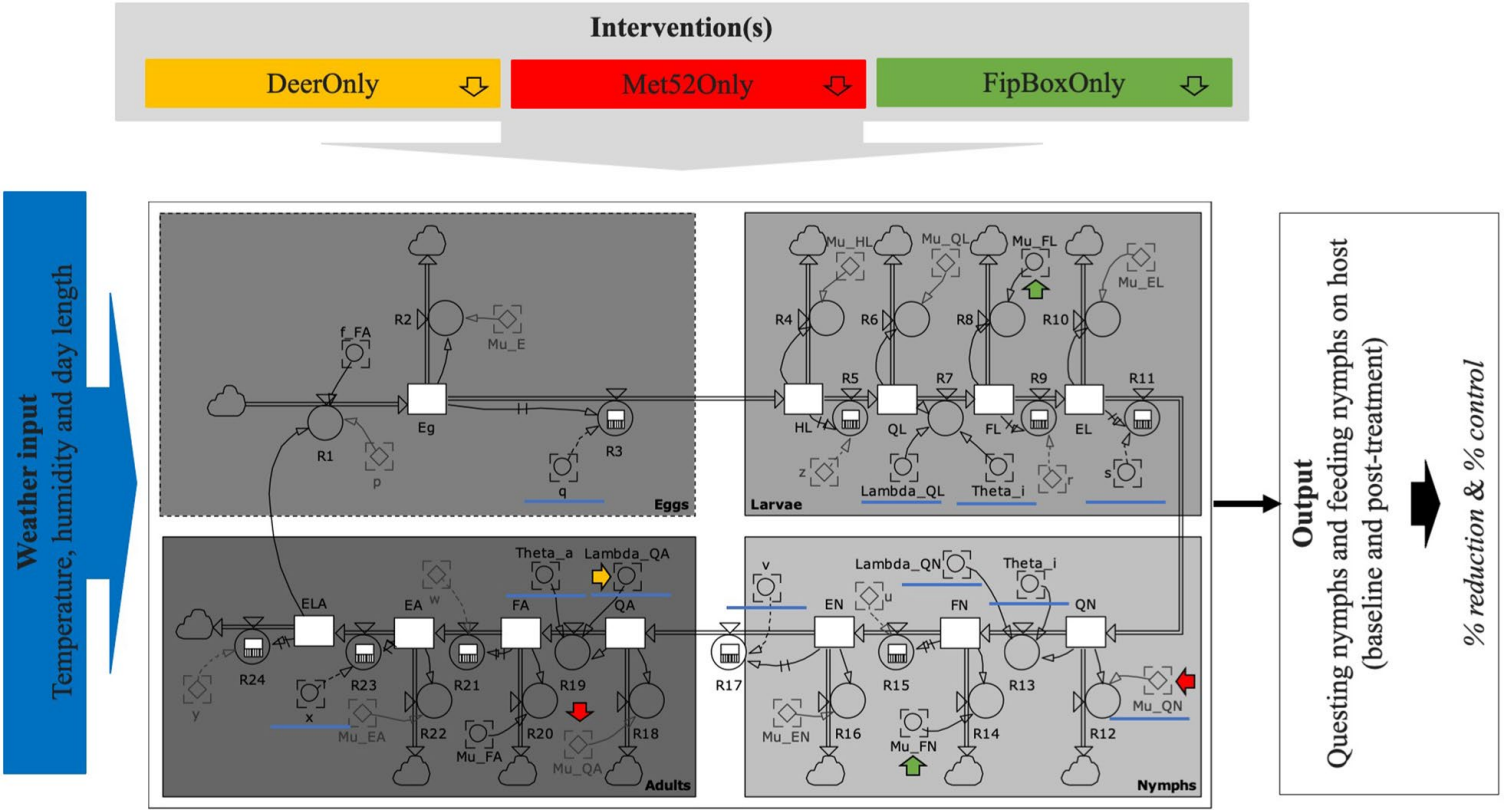
Sketch diagram of the modeling platform. Blue underlines highlight the endogenous variables of the Ogden et al. [31] process-based model that are affected by weather conditions: time delay for the pre-eclosion period of eggs (*q*); host-finding probability for questing larvae, questing nymphs, and questing adults (*λ*_QL_, *λ*_QN_, and *λ*_QA_, respectively); temperature-variable factors for questing activity of immature ticks and adult ticks (*Θ*_*i*_ and *Θ*_*a*_, respectively); time delay for engorged larva to nymph development, or premolt period of engorged larvae (*s*); time delay for engorged nymph to adult development, or premolt period of engorged nymphs (*v*); and time delay for the pre-oviposition period (*x*). The orange, red, and green arrows point to the exogenous and endogenous variables that are affected by the three single interventions (see main text): white-tailed deer population reduction (Deer), broadcast area-application of the entomopathogenic fungus *Metarhizium anisopliae* (Met52), and fipronil-based small rodent bait boxes (FipBox), respectively. A full description of all the parameters of the process-based model is included in the Supplementary material (level variables in Table S2; parameters in Tables S3 and S4; and discrete equations in Table S5)

The platform integrates a process-based mechanistic model with local weather forcing and a set of three tick-reduction interventions (Fig. 1). We calibrated the mechanistic model using data from previously published studies by our group [13, 28] involving residential yards that underwent single or combined tick-reduction interventions, or were included as reference (no intervention) properties (see Supplementary Table S1). First, we calibrated the model parameters related to tick phenology (in absence of intervention) to the data from reference properties. Second, using the data from properties where interventions were implemented, we calibrated more specifically three parameters quantifying the effects of the three tick-reduction interventions on biological mechanisms. Depending on the duration and intensity of the interventions, we modeled them either as “pulse” (instantaneous perturbation of the system, followed by a gradual return to previous state) or “press” interventions (sustained perturbation of the system at constant intensity) [33]. Third, we ran the fully calibrated model multiple times to represent various tick-reduction intervention scenarios and timings. Finally, we analyzed the outcomes of these data-informed model predictions to quantitatively compare the efficacy of these different ITM approaches for the reduction in the DON.

### Process-based model

To represent tick population dynamics, we built an R-version of the Ogden et al. [30] process-based, mechanistic model. The model is based on a system of 12 coupled, nonlinear ordinary differential equations, each representing a given life stage or activity level of the tick population: eggs; hardening, questing, feeding, and engorged larvae; questing, feeding, and engorged nymphs; questing adults; and feeding, engorged, and egg-laying adult females (see Supplementary Tables S2–S5). The mathematical tool was modified to include the weather influence on the mortality (or survival) rate of questing nymphs and the host finding probability, which were both assumed to be constant in the Ogden et al. [30] version of the model.

### Integrated vector control data used for calibration

Data used to calibrate the model were derived from an intervention study previously implemented on residential properties in the town of Redding (41.3044°N, 73.3928°W), Fairfield County, in southwestern Connecticut. Specifically, an ITM program to reduce questing nymphs was conducted from January 2013 through September 2016 [13, 28]. During that period, a total of 41 properties were selected to run the program, including 12 reference (no intervention) properties and 29 properties with implemented interventions. Interventions included single treatments and two- and three-way combinations of three measures, namely white-tailed deer population reduction (Deer), broadcast area-application of the entomopathogenic fungus *Metarhizium anisopliae* (Met52), and fipronil-based small rodent bait boxes (FipBox). Bait boxes were deployed twice at each property during the summer season (May–August) ahead of peak nymphal and larval questing activity, respectively. Sample sizes for each intervention and combination thereof are described in the Supplementary material (Table S1).

### Weather data

To characterize weather conditions, we used three data sources: satellite-gauge combined climatic records (specifically, gridded near-surface air temperature datasets), weather observations, and reanalysis data (specifically, gridded near-surface humidity data). A full description of weather datasets used in simulations is provided in the Supplementary Note 1 and Fig. S1. The model was forced with temperature, humidity, and day length data; the first two variables were corrected to reflect environmental conditions under the leaf litter on the basis of linear transformations from near-surface air temperature and relative humidity to leaf litter temperature and moisture. The linear transformations were determined using data collected at 1 m above ground surface (HOBO U23-001 Pro v2 dataloggers) and in the leaf litter (iButtons) in a previous study (Supplementary Note 1). The conversion aimed to account for ticks spending the majority of their life cycle under leaf litter in forested habitats, where temperature and relative humidity are more moderate than for surrounding air [34, 35]. The adapted Ogden et al. [30] mechanistic model implemented here includes: temperature-dependent development rates for eggs and engorged larvae, nymphs, and females; temperature- and humidity-dependent survival rates of free-living ticks ; temperature-, day length-, and host-density-dependent host-finding rates; and density-dependent survival of all tick stages during engorgement.

### Host data

The 2013–2016 small mammal, live trapping campaign in Redding [13, 28] yielded a grand total of 1298 hosts for immature tick stages captured from residential properties, including white-footed mouse (*Peromyscus leucopus*; 86.4% of captures), eastern chipmunk (*Tamias striatus*; 9.0%), eastern meadow vole (*Microtus pennsylvanicus*; 2.2%), northern short-tailed shrew (*Blarina brevicauda*; 1.9%), American red squirrel (*Tamiasciurus hudsonicus*; 0.2%), and eastern gray squirrel (*Sciurus carolinensis*; 0.1%). We restricted hosts for immature ticks to white-footed mice (as they were the dominant small mammal) and white-tailed deer as the only host for adults in the simulations; both host populations were assumed to be constant.

### Simulating tick population dynamics

The process-based model was run for a time horizon from 1 January 1950 to 31 December 2020 (25,933 days). This long-term period considers an initial 10-year burn-in period for the model to reach equilibrium and additional 10 years to assess day-to-day fluctuations of the tick population after the model had reached stable conditions. The remaining 50 years of the simulation run included the final 2013–2016 experimentation period, for which data are available on the observed densities of questing nymphs expressed per 100 m^2^, from a total of 1244 tick dragging collections conducted over the research period [13, 28].

### Calibration of the model’s tick phenology parameters

Parameters related to tick phenology in the mathematical model were first calibrated to questing nymphal data collected at the reference properties (we averaged the observed questing nymphal data of the 12 properties) by simultaneously minimizing three statistical errors: the percent error (BE) [36], the root mean square error (RMSE) [37], and the general tendency (Pbias) [38] and maximizing the Nash–Sutcliffe efficiency (NSE) [39].

### Efficacy of interventions

We then used data collected from the 29 properties where single or combinations of interventions were implemented (see Table S1) to estimate their efficacy. For a given year at a given property, the Deer treatment was assumed to multiply the host-finding probability for questing adults (*λ*_QA_) between 1 January and 31 December by a factor *f*_D_, where *f*_D_ was calibrated to the data (press function).

We considered that the Met52 treatment affected the per capita mortality rate of questing nymphs (*μ*_QN_) and questing adults (*μ*_QA_) of the process-based model as a pulse (instantaneous perturbation) function. Immediately after the treatment was applied, these two parameters were increased by a factor *f*_M_ (calibrated to the data), followed by a gradual decay in mortality over the effective residual life of Met52, back toward their baseline (without intervention) values at a rate given by:

1 $$1+{f}_{\text{M}}\cdot \text{max}\left(0;1-{\varepsilon }^{\frac{28-\Delta t}{14}}\right)$$

where *Δt* depicts the number of days since the treatment application and where *ε* is a tuning parameter set to 0.1. *ε* is a tuning parameter that allows the effect of treatment on mortality to decrease over time, as shown in Supplementary Fig. S2 (upper panel). From formula (1), ε affects both the strength of the effect at treatment application (that is, *f*_M_ (1 − *ε*^2^) at *Δt* = 0) and the shape of the decrease or derivative (that is, *f*_M_ *ε*^(28− *Δt*)/14^ log(*ε*)/14). Therefore, *ε* cannot exactly be set to 0 but needs to be close to 0 to obtain an effect value close to *f*_*M*_ at *Δt* = 0. This is why we arbitrarily set it to 0.1. The effective residual life of Met52 was assumed to be 4 weeks (or 28 days) [40]. The first and second application rounds took place in early June and early July respectively, leading to an impact of Met52 only treatment lasting for two consecutive months (early June to early August), consistent with the broadcast applications during the 2013–2016 control campaign.

Similarly, the FipBox treatment was assumed to impact the per capita mortality rate of feeding larvae (*μ*_FL_) and feeding nymphs (*μ*_FN_) of the process-based model as a pulse function (see Fig. S2). These two parameters were increased by a factor *f*_F_ (calibrated to the data) after boxes were deployed, followed by a gradual decay in mortality over the effective residual life of the compound, back toward their baseline (without intervention) values at a rate given by:

2 $$1+{f}_{\text{F}}\cdot \text{max}\left(0;1-{\varepsilon }^{\frac{56-\Delta t}{28}}\right)$$

where *Δt* depicts the number of days since the treatment application and *ε* is a tuning parameter set to 0.1. The effective residual life of the compound was assumed to be 8 weeks (or 56 days). To account for two deployments of bait boxes during the campaign, we simulated an increase in tick mortality in early May and again after 8 weeks (i.e., approximately 16 weeks in total), the period after which bait boxes were retrieved.

Parameters *f*_D_, *f*_M_, and *f*_F_ were simultaneously estimated by minimizing the RMSE for all 29 properties with interventions, which enabled assessment of the effect of each individual intervention despite the simultaneous implementation in some of the properties.

### Simulation of the effectiveness of ITM approaches

Using the values of *f*_D_, *f*_M_, and *f*_F_ estimated from the data, we simulated the implementation of all combinations of the three treatments: Deer only (D), Met52 only (M), FipBox only (F), Deer + Met52 (D + M), Deer + FipBox (D + F), Met52 + FipBox (M + F), and Deer + Met52 + FipBox (D + M + F).

### Simulation of alternative number and timing of interventions

The last set of simulations included the analysis of changes in the Met52 only treatment applications. Starting from the baseline scenario with two applications of treatment in early June and early July, respectively, we assessed the effects of shifting the timing of application and reducing the number of applications. We simulated a change in the dates of both Met52 only applications: the first application taking place between late April and mid-July, and the second application 29 days later. We also simulated these alternatives in a scenario where only the first application of Met52 only was performed.

### Metrics for the percent reduction

In these different scenarios, we calculated the percent reduction of the predicted (simulated) nymphal peak, i.e., the percent reduction in the number of feeding nymphs per host at the peak of each nymphal season associated with each single treatment and ITM approaches. We also calculated the percentage change in the area under the phenology curve, which accounts for the seasonal variation in the densities of feeding nymphs compared with reference plots, for simulated single treatments and combined efforts.

## Results

### Model calibration

**Fig. 2:**
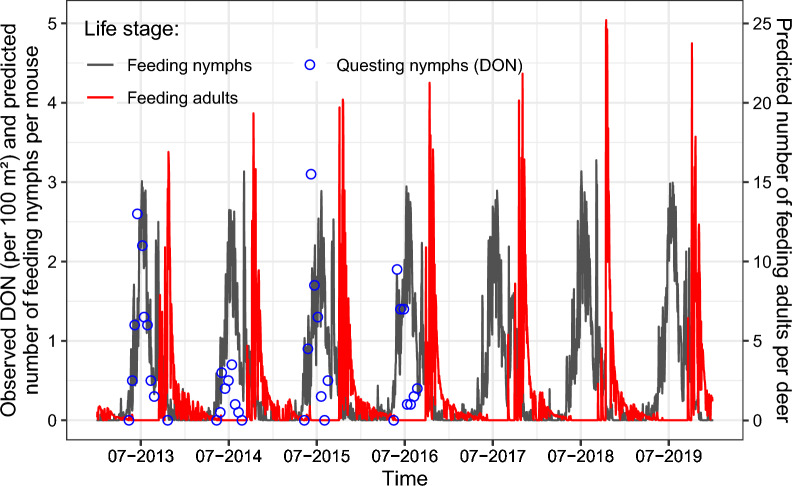
Daily time series of feeding ticks on host simulated by the dynamic tick population model for the historical period 2013–2019, for in situ conditions corrected to reflect temperature and moisture conditions under leaf-litter, for weather-dependent host finding probability, and for the group of properties included in ITM reference. Black and red time series depict the simulation outputs when the model is forced with near-surface air temperature weather station data. The blue dots depict the average number of observed densities of nymphs per 100 m^2^

For all reference properties (ITM reference), simulation outputs of the process-based model yielded a BE value of 29.1%, a RMSE value of 1.12, a Pbias value of −64.9, and a NSE value of −0.98, when the model was forced with near-surface air temperature weather station data. Predictions of the calibrated model are shown in Figs. 2 and S3. In the absence of interventions, the model predicted yearly feeding nymphal peaks at around three nymphs per mouse (Figs. 2 and S3).

### Efficacy of interventions on reduction of the peak in feeding nymphs

**Fig. 3:**
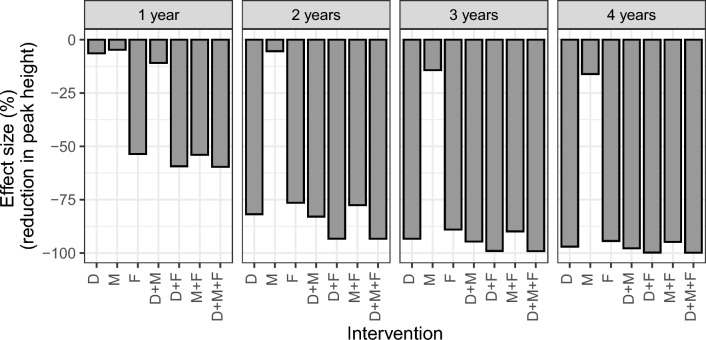
Percent reduction of the height of the feeding nymphal peak on the last year of intervention, compared with the no intervention scenario. For each intervention (single and combined), different alternatives for the number of years of implementation (from 1 to 4 years) are presented. D: Deer only; M: Met52 only; F: FipBox only; D + M: Deer + Met52; D + F: Deer + FipBox; M + F: Met52 + FipBox; D + M + F: Deer + Met52 + FipBox

**Fig. 4:**
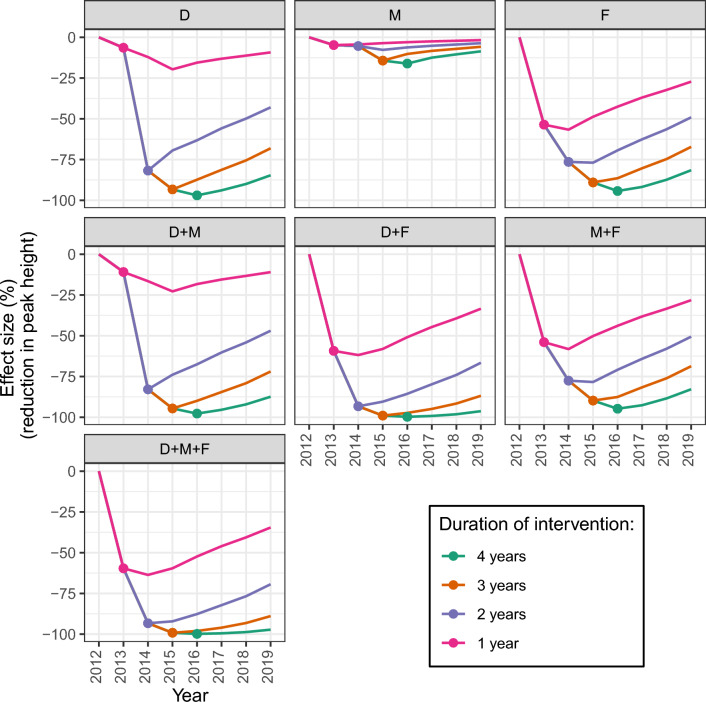
Evolution of the percent reduction of the height of the feeding nymphs annual peak for each intervention, compared with the no intervention scenario. In our model simulations, interventions are all implemented in 2013, for 1 to 4 years. For each intervention and number of years, the dot represents the last year of intervention, with the line representing changes in effect size with years after the intervention ended. D: Deer only; M: Met52 only; F: FipBox only; D + M: Deer + Met52; D + F: Deer + FipBox; M + F: Met52 + FipBox; D + M + F: Deer + Met52 + FipBox

The point estimate for *f*_M_ was 1.25, meaning that the Met52 only treatment was estimated to multiply the daily mortality of questing nymphs and adults by 2.25 on the first day of application, an effect then decreasing with time (Eq. 1). The point estimate for *f*_F_ was 4.11, meaning that the FipBox treatment was estimated to multiply the daily mortality of feeding larvae and nymph by 5.11 on the first day, an effect then decreasing with time (Eq. 2) (Fig. S2). The point estimate for *f*_D_ was 0.985, meaning that the Deer only intervention was estimated to reduce the host-finding rate for adults in yards by 98.5%. In model simulations utilizing these parameters, the efficacy of all interventions was higher and persisted for a longer time (Figs. 3, 4 and S4) when they were applied for a higher number of consecutive years. Combinations of multiple interventions were always more efficacious than single interventions (Fig. 3).

Among single interventions implemented for only 1 year, FipBox only was the most efficacious as it decreased the feeding nymphal peak on that year by 53.6% compared with the no intervention scenario (by killing the nymphs feeding on hosts), versus 6.4% and 4.7% for the Deer only and Met52 only, respectively (Fig. 3). However, because the FipBox treatment affects both larvae and nymphs on host, it showed its peak efficacy on the nymphal population the year following the first year of intervention (56.7% reduction of the nymphal peak) (Fig. 4). Similarly, because of its effects on adult ticks, the Deer only intervention reached its peak efficacy 2 years after the first year of intervention, with a 19.6% decrease in nymphal peak (Figs. 4 and S4).

When implemented for several years, Deer only reached a similar efficacy to FipBox only, i.e., a 97.0% versus 94.3% reduction, respectively, of the nymphal peak on the last year of 4 years of intervention, compared with the no intervention scenario. The Met52 only treatment was less efficacious even after 4 years of implementation (16.1% reduction) (Figs. 3, 4).

The combination of the three interventions (Deer + Met52 + FipBox) was always the most efficacious, leading to a decrease of the nymphal peak of 59.6% (after 1 year of implementation) to 99.8% (after 4 years of implementation), compared with the no intervention scenario. However, it was closely followed by the Deer + FipBox intervention, whose efficacy was never more than 1% lower than the Deer + Met52 + FipBox scenario. Assuming four consecutive years of implementation, all interventions (single and combined) reached a reduction in the nymphal peak of more than 94% compared with no intervention, except Met52 only (Figs. 3, 4).

### Comparison of percent reduction metrics

The results described above were qualitatively similar when we considered the reduction in the area under the feeding nymphs phenology curve instead of the height of the feeding nymphal peak (Figs. S5 and S6).

### Changes in the timing of Met52 only interventions

**Fig. 5:**
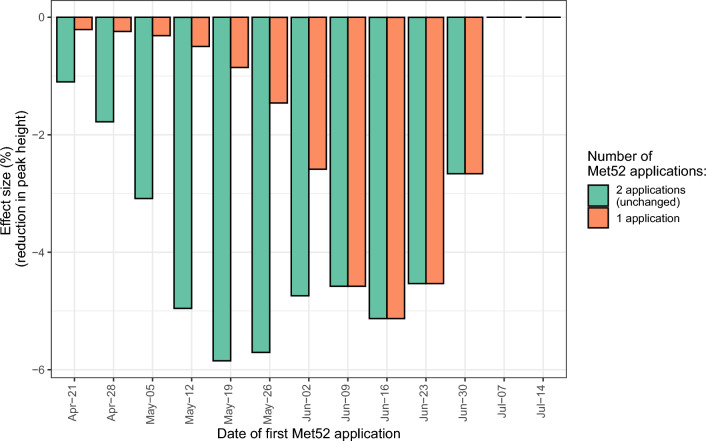
Percent reduction of the height of the feeding nymphal peak in the only year of intervention, compared with the no intervention scenario, for different variations of the Met52 only treatment. One or two Met52 applications are simulated, starting at different dates between 12 April and 14 July. If done, the second Met52 application always takes place 29 days after the first one. The application of Met52 after the nymphal peak (here, after 7 July) has, as expected, no impact on peak height

From our model simulations, the application of the Met52 only treatment on 19 May followed by a second application 29 days later led to the highest reduction of the nymphal peak that year (− 5.85% compared with the no intervention scenario) (Fig. 5). However, applying the Met52 only treatment only once in mid-June led to almost similar results (−5.13% compared with the no intervention scenario) while reducing the amount of input needed (Fig. 5). The advantage of applying a second Met52 only treatment was different when measuring the area under the phenology curve (Fig. S7), with a maximum effect size of −3.61% for two treatments, instead of −1.98% for one treatment, compared with the no intervention scenario.

## Discussion

We found that combined treatments were globally more efficacious than single treatments in reducing DON  in residential yards. Among single treatments, our results suggest that deer reduction (Deer) and fipronil-based small rodent bait boxes (FipBox) were more efficacious than the broadcast area-application of the entomopathogenic soil-borne fungus *Metarhizium anisopliae* (Met52). Because interventions targeted different tick life stages, they impacted the nymphal tick outcome at different time lags, with an immediate effect of Met52, an effect of FipBox reaching its maximum after a 1-year lag, and deer reduction 2 years after the intervention [17]. Globally, higher number of subsequent years of (single or combined) interventions led to better efficacy. Most interventions (except Met52 only) reached a reduction in the feeding nymph peak of more than 94% compared with the no intervention scenario when implemented for at least 4 years. Moreover, we found that the optimal date for a single application of Met52 in Connecticut residential yards would be around mid-June. Preceding this application by another application 1 month earlier (in mid-May) would only slightly reduce the height of the feeding nymph peak (maximum exposure potential) but would reduce by around half the area under the phenology curve across the whole season. The area under the phenology curve is a cumulative  measure of human exposure risk because exposure to questing nymphs can occur throughout the period of nymphal activity (results shown in the supplement).

Previous empirical studies investigated the effect of interventions on the DON, tick burden on hosts, or the prevalence of tick infection by various pathogens [13, 17, 18, 22, 28, 41–43]. Some published models also theoretically simulated the implementation of interventions (vaccination) in a tick–host system [44, 45]. Our study combined both of these approaches by using data collected from field experiments to estimate intervention effect parameters in a mechanistic model. This method is innovative for tick–host systems, while more common for other vector-borne disease systems [46–48], and has several advantages. First, it provides a mechanistic and dynamic understanding of interventions, in contrast with empirical-only studies, which only assess differences in global output (e.g., the density of questing nymphs at a single point in time). For example, we estimated that Met52 only in our study multiplied the daily mortality rate of questing nymphs and adults by 2.25 on the first day of application and could estimate the cumulative impact of the intervention during multiple years. Second, once mechanistic intervention effect parameters are calibrated to the data, it is possible to explore a wide range of simulated treatment scenarios in terms of timing or combinations of interventions. Simulations are hence easier than implementing a large number of treatment options in the field, and can narrow down which combinations, intensity, and timing of interventions should be further tested in experimental studies.

The effect sizes identified in our model were consistent with some previous studies. On the year of intervention, we predicted similar reductions of the number of feeding nymphs per mouse (nymphal burden) than a previously published empirical study [49] for both FipBox (around 50%) and Met52 (less than 15%, not significant in the empirical study). Our results were also consistent with Ostfeld et al. [42], who found a stronger effect of FipBox than Met52 on the nymphal burden on mice.

In our study, despite the fact that the deer population was not entirely removed [28], we predicted a strong effect of this intervention on the nymphal peak when it was implemented for several years. However, our modeling framework did not include the infection process, and the effect of deer population reduction (rather than elimination) on nymph infection prevalence and the density of infected nymphs is still unclear [50, 51]. Immediately following deer reduction, in the absence of numerous large-bodied hosts, sampling efforts for remaining questing ticks often result in a perceived, temporary amplification in both abundance and pathogen infection prevalence as juveniles engaging in questing behavior and are then more likely to obtain a bloodmeal from a reservoir-competent host [22, 50–52].

Our study has some limitations. First, Gaff et al. [53] found that assuming constant instead of varying host population densities—as we did—can affect the density of nymphs predicted by a model, although it hardly changes the predicted number of feeding ticks per host, which was the output we considered here. Second, we did not incorporate host movements in our model as in Li et al. [54], Wang et al. [55] and Wang et al. [56]. In the future, adding host behavioral components to the model would more directly describe human exposure to tick-borne disease hazard. Moreover, expanding the model to include infection dynamics and other sets of interventions, including vaccines and reductions in tick habitat suitability through landscape and vegetation management, will allow for estimates of efficacy in reducing the density of infected nymphs. Integrating the costs of interventions would allow for a more thorough cost-effectiveness analysis of tick- and host-targeted interventions in residential yards [57].

## Conclusions

Computer simulations allow us to identify optimal control targets to minimize exposure to tick bites and the risk for tick-borne diseases. Our long-term goal is to provide information to stakeholders on the optimal deployment of individual and combined tick management approaches that can synergistically contribute to short-to-long-term, cost-effective, and sustainable control of tick vectors using integrated vector management guidelines.

### Supplementary Information

Additional file 1.

## Data Availability

The data that support the findings of this study are available from the corresponding author upon reasonable request. All images were generated by the authors, so no consent for publication was required.
