# Supplementary material for: Modeling platform to assess the effectiveness of single and integrated Ixodes scapularis tick control methods
Source: Parasit Vectors. 2024 Aug 12;17:339. doi: 10.1186/s13071-024-06387-2 (PMC11321154; doi:10.1186/s13071-024-06387-2)
Supplement: Supplementary file 1 — Additional file 1. [file 13071_2024_6387_MOESM1_ESM.docx]

**Modeling platform to assess the effectiveness of single and integrated *Ixodes scapularis* tick control methods**

**Supplementary material**

Daniel Ruiz-Carrascal, Jonathan Bastard, Scott C. Williams, and Maria Diuk-Wasser

Single “Deer Only” treatments were conducted in eight (8) properties. A single “FipBox Only” was deployed in one (1) property. A combination of Met52+FipBox was conducted in thirteen (13) properties, but in 2016 only FipBox were deployed. A combination of Deer+Met52+FipBox was conducted in five (5) properties, but in 2016 a combination of Deer+FipBox was conducted. A combination of Deer+FipBox was conducted in two (2) properties. Lastly, twelve (12) properties were used as experimental reference (i.e., controls) and received no intervention (Integrated Tick Management – ITM Reference).

***Table S1.*** Assigned treatments to residential properties (n=41) over four years as part of an integrated tick management program conducted in Redding.

| **#** | **Assigned treatment** | **2013** | **2014** | **2015** | **2016** |
| --- | --- | --- | --- | --- | --- |
| 1 | Deer Only | Yes | Yes | Yes | $, £ |
| 2 | Deer Only | Yes | Yes | Yes | Yes |
| 3 | Deer Only | Yes | Yes | Yes | Yes |
| 4 | Deer Only | Yes | Yes | Yes | Yes |
| 5 | Deer Only | Yes | Yes | Yes | $ |
| 6 | Deer Only | X | Yes | Yes | Yes |
| 7 | Deer Only | X | Yes | Yes | $ |
| 8 | Deer Only | X | Yes | Yes | $ |
| 9 | ITM Reference | Yes | Yes | Yes | Yes |
| 10 | ITM Reference | Yes | Yes | Yes | Yes |
| 11 | ITM Reference | Yes | Yes | Yes | Yes |
| 12 | ITM Reference | Yes | Yes | Yes | Yes |
| 13 | ITM Reference | Yes | Yes | Yes | Yes |
| 14 | ITM Reference | Yes | Yes | Yes | Yes |
| 15 | ITM Reference | X | Yes | Yes | Yes |
| 16 | ITM Reference | X | Yes | Yes | Yes |
| 17 | ITM Reference | X | Yes | Yes | Yes |
| 18 | ITM Reference | X | Yes | Yes | Yes |
| 19 | ITM Reference | X | Yes | Yes | Yes |
| 20 | ITM Reference | X | Yes | Yes | Yes |
| 21 | Met52+FipBox and FipBox Only | Yes | Yes | Yes | FipBox Only |
| 22 | Met52+FipBox and FipBox Only | Yes | Yes | Yes | $, £ |
| 23 | Met52+FipBox and FipBox Only | Yes | Yes | Yes | FipBox Only |
| 24 | Met52+FipBox and FipBox Only | Yes | Yes | Yes | FipBox Only |
| 25 | FipBox Only | $ | $ | $ | Yes |
| 26 | Met52+FipBox and FipBox Only | Yes | Yes | Yes | FipBox Only |
| 27 | Met52+FipBox and FipBox Only | Yes | Yes | Yes | FipBox Only |
| 28 | Met52+FipBox and FipBox Only | X | Yes | Yes | FipBox Only |
| 29 | Met52+FipBox and FipBox Only | X | Yes | Yes | FipBox Only |
| 30 | Met52+FipBox and FipBox Only | X | Yes | Yes | FipBox Only |
| 31 | Met52+FipBox and FipBox Only | X | Yes | Yes | FipBox Only |
| 32 | Met52+FipBox and FipBox Only | X | Yes | Yes | FipBox Only |
| 33 | Met52+FipBox and FipBox Only | X | Yes | Yes | FipBox Only |
| 34 | Met52+FipBox and FipBox Only | X | Yes | Yes | FipBox Only |
| 35 | Deer+Met52+FipBox (Everything) | Yes | Yes | Yes | Deer+FipBox |
| 36 | Deer+Met52+FipBox (Everything) | Yes | Yes | Yes | Deer+FipBox |
| 37 | Deer+Met52+FipBox (Everything) | Yes | Yes | Yes | Deer+FipBox |
| 38 | Deer+FipBox | Yes | Yes | Yes | $ |
| 39 | Deer+FipBox | Yes | Yes | Yes | $ |
| 40 | Deer+Met52+FipBox (Everything) | Yes | Yes | Yes | Deer+FipBox |
| 41 | Deer+Met52+FipBox (Everything) | X | Yes | Yes | Deer+FipBox |

Deer: White-tailed deer reduction; FipBox: Fipronil-based small rodent bait boxes; Met52: Broadcast area-application of the entomopathogenic fungus *Metarhizium anisopliae*; ITM Reference: Integrated Tick Management Reference.

$ Not sampled; £ Withdrew

***Table S2.*** Level or state variables of the Ogden et al. (2005) process-based, mechanistic model.

| **Life stage** | **Activity** | **Description** |
| --- | --- | --- |
| Eggs | -- | Number of eggs, Eg(t); Eg(0) = 0 |
| Larvae | Hardening | Number of hardening larvae, H_L_(t); H_L_(0) = 0  This level variable comprises hatched larvae undergoing a 21 day 'hardening' prior to becoming questing larvae. |
|  | Questing | Number of questing larvae, Q_L_(t); Q_L_(0) = 0 |
|  | Feeding | Number of feeding larvae, F_L_(t); F_L_(0) = 0 |
|  | Engorging | Number of engorged larvae, E_L_(t); E_L_(0) = 0 |
| Nymphs | Questing | Number of questing nymphs, Q_N_(t); Q_N_(0) = 0 |
|  | Feeding | Number of feeding nymphs, F_N_(t); F_N_(0) = 0 |
|  | Engorging | Number of engorged nymphs, E_N_(t); E_N_(0) = 0 |
| Adults | Questing | Number of questing adults, Q_A_(t); Q_A_(0) = 10,000 |
|  | Feeding | Number of feeding adult females, F_A_(t); F_A_(0) = 0 |
|  | Engorging | Number of engorged adult females, E_A_(t); E_A_(0) = 0 |
|  | Egg-laying | Number of egg-laying adult females, EL_A_(t); EL_A_(0) = 0 |

***Table S3.*** Eggs- and larvae-related parameters of the Ogden et al. (2005) process-based, mechanistic model.

| **Life stage** | **Description** |
| --- | --- |
| Eggs-related | Per-capita egg production by egg-laying females (p). Note: in the original version of the model, the maximum number of eggs produced by each engorged adult female was set to 3,000, following the suggestions by (Mount et al.,1997). In our revised version, p was set to 350 eggs. |
|  | Daily, per-capita mortality rate of eggs (μ_E_). Set to 0.0020. |
| Larvae-related | Time delay for hardening of larvae (z). Set to 23 days. |
|  | Time delay for the feeding period of larvae (r). Ticks feed for a fixed time delay, after which they instantaneously *drop off* the host as engorged ticks. Set to 3 days. |
|  | Daily, per-capita mortality rate of hardening larvae (μ_HL_). Set to 0.0060. |
|  | Daily, per-capita mortality rate of questing larvae (μ_QL_). Set to 0.0060. |
|  | Daily, per-capita mortality rate of engorged larvae (μ_EL_). Set to 0.0030. |

***Table S4.*** Nymphs-, adults- and hosts-related parameters of the Ogden et al. (2005) process-based, mechanistic model.

| **Life stage** | **Description** |
| --- | --- |
| Nymphs-related | Time delay for the feeding period of nymphs (u). Set to 5 days. |
|  | Daily, per-capita mortality rate of questing nymphs (μ_QN_). Note: two approaches are proposed here: (i) set μ_QN_ to a constant value of 0.0060; or (ii) make the parameter dependent on the survival rate of questing nymphs, *SR_QN_*, which is given by (Gaff et al., 2020):  ${SR}_{QN}=\left[ \frac{0.999+0.02088 T-0.00137 T^{2}}{1+0.02094 T-0.00136 T^{2}} \right] \left( -7.40667\cdot{10}^{-5} {sd}^{2}+4.443333\cdot{10}^{-4} sd+0.999 \right)$,  where *T* represents the daily near-surface mean air temperature, expressed in ℃, and *sd* the daily saturation deficit, expressed in mb. |
|  | Daily, per-capita mortality rate of engorged nymphs (μ_EN_). Set to 0.0020. |
| Adults-related | Time delay for the feeding period of adult females (w). Set to 10 days. |
|  | Time delay for oviposition (y). Set to a fixed time delay of 1 day, but it varies with temperature in nature. |
|  | Daily, per-capita mortality rate of questing adults (μ_QA_). Set to 0.0060. |
|  | Daily, per-capita mortality rate of engorged adults (μ_EA_). Set to 0.0001. |
| White-footed mice-related | Number of rodents (Rod) (hosts for immature ticks). Set to 250 rodents. |
| White-tailed deer-related | Number of deer (D) (hosts for adult ticks). Set to 20 deer. |

***Table S5.*** Dynamics of the Ogden et al. (2005) process-based, mechanistic model’s level variables.

| **Level variable** | **Dynamic equation** |
| --- | --- |
| Eg(t) | $\frac{dEg(t)}{dt}={EL}_{A}\left( t \right).f_{FA}\left( t \right).p-\mu_{E}.Eg\left( t \right)-\frac{Eg(t)}{q(t)}$ |
| H_L_(t) | $\frac{dH_{L}(t)}{dt}=\frac{Eg(t)}{q(t)}-\mu_{HL}.H_{L}\left( t \right)-\frac{H_{L}(t)}{z}$ |
| Q_L_(t) | $\frac{dQ_{L}(t)}{dt}=\frac{H_{L}(t)}{z}-\mu_{QL}.Q_{L}\left( t \right)-\lambda_{QL}.Q_{L}\left( t \right).\Theta_{i}\left( t \right)$ |
| F_L_(t) | $\frac{dF_{L}(t)}{dt}=\lambda_{QL}.Q_{L}\left( t \right).\Theta_{i}\left( t \right)-\mu_{FL}(t).F_{L}\left( t \right)-\frac{F_{L}(t)}{r}$ |
| E_L_(t) | $\frac{dE_{L}(t)}{dt}=\frac{F_{L}(t)}{r}-\mu_{EL}.E_{L}\left( t \right)-\frac{E_{L}(t)}{s(t)}$ |
| Q_N_(t) | $\frac{dQ_{N}(t)}{dt}=\frac{E_{L}(t)}{s(t)}-\mu_{QN}.Q_{N}\left( t \right)-\lambda_{QN}.Q_{N}\left( t \right).\Theta_{i}\left( t \right)$ |
| F_N_(t) | $\frac{dF_{N}(t)}{dt}=\lambda_{QN}.Q_{N}\left( t \right).\Theta_{i}\left( t \right)-\mu_{FN}(t).F_{N}\left( t \right)-\frac{F_{N}(t)}{u}$ |
| E_N_(t) | $\frac{dE_{N}(t)}{dt}=\frac{F_{N}(t)}{u}-\mu_{EN}.E_{N}\left( t \right)-\frac{E_{N}(t)}{v(t)}$ |
| Q_A_(t) | $\frac{dQ_{A}(t)}{dt}=\frac{E_{N}(t)}{v(t)}-\mu_{QA}.Q_{A}\left( t \right)-\lambda_{QA}.\frac{Q_{A}\left( t \right)}{2}.\Theta_{a}\left( t \right)$ |
| F_A_(t) | $\frac{dF_{A}(t)}{dt}=\lambda_{QA}.\frac{Q_{A}\left( t \right)}{2}.\Theta_{a}\left( t \right)-\mu_{FA}(t).F_{A}\left( t \right)-\frac{F_{A}(t)}{w}$ |
| E_A_(t) | $\frac{dE_{A}(t)}{dt}=\frac{F_{A}(t)}{w}-\mu_{EA}.E_{A}\left( t \right)-\frac{E_{A}(t)}{x(t)}$ |
| EL_A_(t) | $\frac{d{EL}_{A}(t)}{dt}=\frac{E_{A}(t)}{x(t)}-\frac{{EL}_{A}(t)}{y}$ |

The host finding probability for questing larvae (*λ_QL_*) and questing nymphs (*λ_QN_*) (i.e., questing immature ticks) is given by:

$\lambda_{QL}=\lambda_{QN}= 0.010 R^{0.515}\left( -0.0105 T^{2}+0.4316 T-3.424 \right)\left[ \frac{0.03116-0.007615 DL+0.0004469 {DL}^{2}}{1-0.1374 DL+0.004788 {DL}^{2}} \right]^{2}$,

for 10.8<T<30.2, sd<8.0 and DL>DL_c_, where DL depicts the daily day length, and DL_c_ the critical day length, or the difference between the maximum day length and the day-to-day standard deviation of day length. In the equation, R depicts the total number of rodents (hosts for immature ticks).

The host finding probability for questing adults (*λ_QA_*) (i.e., questing mature ticks) is given by:

$\lambda_{QA}=0.050 D^{0.515}\left( -0.0095 T^{2}+0.19 T+0.05 \right)$,

for 0.0<T<20.2. In this equation, D depicts the total number of deer (hosts for adult ticks).

***Supplementary Note 1.***

*Weather.* Large-scale climate data were downscaled to the local scale and day-to-day variability was added.

Gridded data. We downloaded the NOAA NCEP CPC GHCN CAMS (Fan and van den Dool, 2004; 2008) and UCSB CHIRTS v1.0 (Funk et al., 2019) gridded near-surface air temperature datasets from the IRI Data Library. This dataset (CAMS) is available at a spatial resolution of 0.5º (~56 km) and at a monthly timescale for the continuous period spanning from January, 1948 to present. The CHIRTS dataset is available at a spatial resolution of 0.05º (~6 km) and at a daily timescale, and its historical period spans from January 1, 1983 to December 31, 2016. These datasets were restricted to the geo-domain 40.47 to 41.53 N, and 74.58 to 71.81 W; i.e., a bounding box in the vicinity of Redding. Within this geo-domain we selected a single gridpoint of the CAMS and CHIRTS gridded datasets where the town of Redding (41.3044°N, 73.3928°W) is located. Such a gridpoint encompasses all the properties in the study site. The CAMS and CHIRTS datasets were averaged to have a continuous daily time series of mean temperatures for the historical period January 1, 1950 to present. Because the CHIRTS gridded dataset only spans the historical period 1983-2016, and the process-based model needs to be forced with climate data spanning the period 1950-2020 (i.e., the model's time horizon), weather data for the years 1950-1982 and 2017-2020 result in the same monthly values of the CAMS gridded dataset; i.e., the averaging is only applied for the historical period 1983-2016.

Weather station data. We used the set of daily observations gathered at the station USW00054734 Danbury Municipal Airport, which is located at 41.37215°N, 73.48337°W and 138 masl. Records were downloaded from the Climate Data Online portal (https://www.ncdc.noaa.gov/cdo-web/) of the National Oceanic and Atmospheric Administration's National Centers for Environmental Information (daily summaries are available at: https://www.ncei.noaa.gov/maps/daily-summaries/). The instrumental period of this weather station spans from May 11, 1998 to present.

Bias adjustment. We corrected the daily averaged CAMS and CHIRTS gridded data using bias adjustment with the weather station (point estimate) data. We first extracted the gridded data at the exact location of the weather station, and the series (station data and gridded dataset) were used to compute bias factors. We quantified the bias of the gridded data at the exact location of the weather station, assuming the station data is accurate. Since we are using daily data, we centered a 5-day time window to account for day-to-day variability. We quantified bias factor for day t at a station j, and then assume a spatially heterogeneous bias (independent and normally distributed). The bias factor at the exact location of our weather station was interpolated to the gridded data and then used to adjust or correct the gridded data. The corresponding adjusted value was obtained by multiplying the gridded data with the bias factor. A comparison between the CAMS monthly temperature, the CHIRTS daily temperature historical time series, the daily averaged CAMS and CHIRTS gridded time series, the NOAA NCEI CDO daily weather station data, and the bias-corrected gridded average for a single year (2016) is shown in the upper panel of figure S1. The latter time series is depicted by the blue solid line.

We downloaded specific humidity data (at 1,000 mb) from the publicly available 0.75º-arc global atmospheric ERA-5 reanalysis data of the European Center for Medium-Range Weather Forecasts - ECMWF (Potter et al., 2018). To calculate saturation deficit, specific humidity records were combined with sea level pressure and near-surface air temperature data. ERA-5 datasets are available for the historical period spanning from January 1, 1979 to August 31, 2019. The geo-domain of interest was also restricted to 40.47 to 41.53 N, and 74.58 to 71.81 W. Lastly, we calculated the daily day length of Redding using the sunrise and sunset times available on the Global Radiation Group portal of the NOAA Global Monitoring Laboratory at (https://gml.noaa.gov/grad/solcalc).

The process-based model was forced with bias-adjusted daily-averaged CAMS and CHIRTS satellite-gauge combined gridded near-surface air temperature datasets to compute in-situ (1 m above the surface) near-surface mean air temperature and daily air temperature under leaf litter. We used linear transformations derived from a previous study conducted by our team on Block Island (Town of New Shoreham), off the coast of Rhode Island. In such study, we installed HOBO U23-001 Pro v2 Temperature/Relative Humidity data loggers in grid dragging sites at an altitude of 1 m above ground surface, and gathered data every 30 minutes continuously over the period 2013-2020. iButton devices were installed in each of the grid sites, close to the HOBO data loggers and right below the leaf litter. Due to their limited memory, iButtons gathered data continuously every 30 minutes only during the field sampling seasons (~May to August) of the period 2013-2017. To transform gridded datasets or weather station data into in-situ near-surface mean air temperature data, we used the linear relationship c_1_*DailyT+c_2_, where c_1_ and c_2_ depict the slope parameter and the intercept of datalogger versus gridpoint air temperature data, respectively. Daily 1-m near-surface air mean temperature data were corrected to compute daily air temperature data under leaf litter by using the linear relationship c_3_*DailyT+c_4_, where c_3_ and c_4_ depict the slope parameter and the intercept of leaf litter versus datalogger air temperature data, respectively. ERA-5 reanalysis saturation deficit data were corrected to get in-situ, under leaf litter moisture conditions by using the linear relationship c_5_*DailySD+ c_6_, where c_5_ and c_6_ depict the slope parameter and the intercept of local weather station saturation deficit data versus ERA-5 gridded saturation deficit data, respectively. The following parameter values were assumed in our study: c_1_=0.9346; c_2_=1.0183; c_3_=0.8058; c_4_=4.9378; c_5_=0.5760; and c_6_=1.0589. In the middle panel of figure S1, a comparison between NOAA NCEI CDO weather station air temperature data and the satellite-gauge gridded air temperature dataset is shown. In the lower panel of the same figure, linear transformations that were derived from our previous study are shown. In the left panel, a comparison between daily near-surface air temperatures provided a local NOAA NCEI weather station (x-axis) and the corresponding daily records gathered with HOBO U23-001 Pro v2 Temperature/Relative Humidity data loggers (y-axis) is shown. The instrumental period is restricted to June 1, 2013 to December 31, 2019. In the right panel, a comparison between daily near-surface air temperature gathered with HOBO U23-001 Pro v2 Temperature/Relative Humidity data loggers (x-axis) and the corresponding readings gathered with iButtons (leaf litter temperatures; y-axis) is shown. The instrumental period is restricted to June 1, 2013 to December 31, 2019. The longest historical periods within the instrumental period above are considered to calculate the main statistics (shown in each of the panels) of the time series.


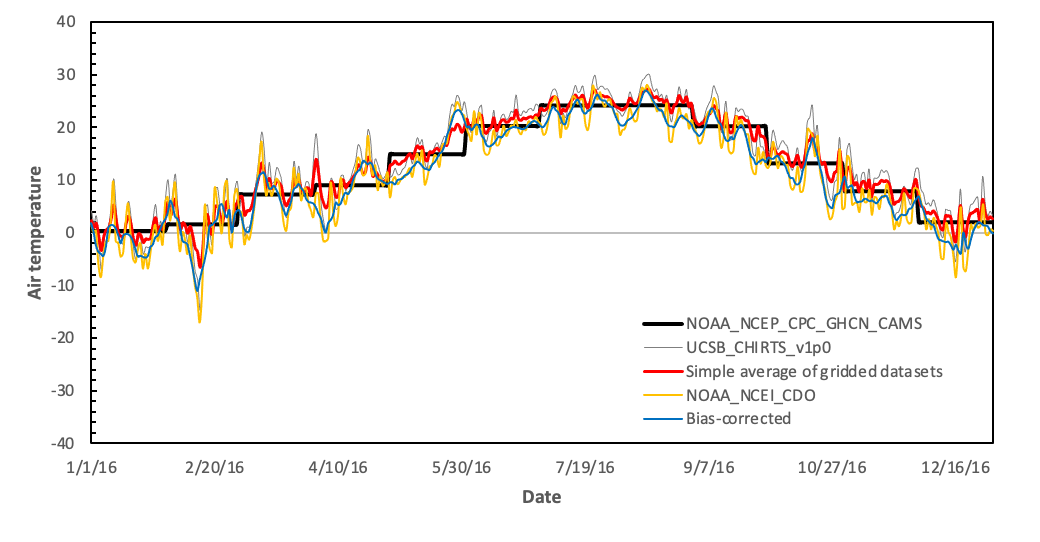


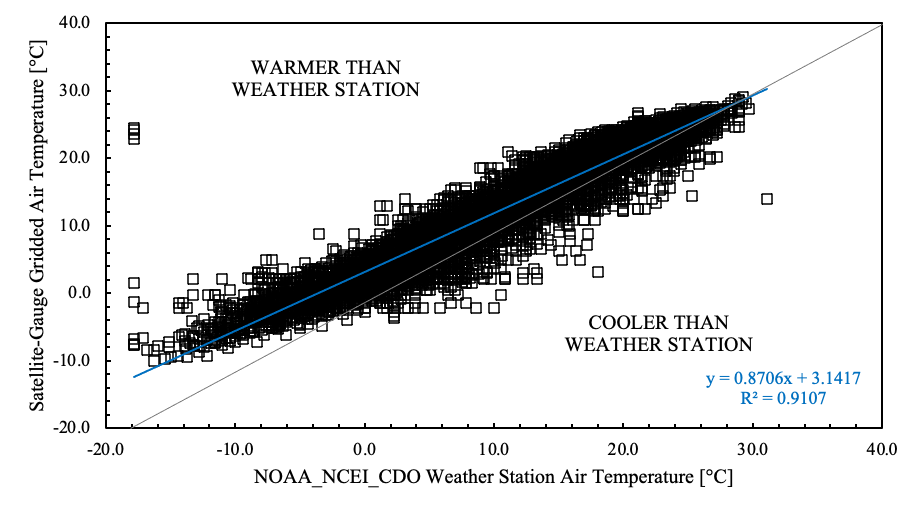


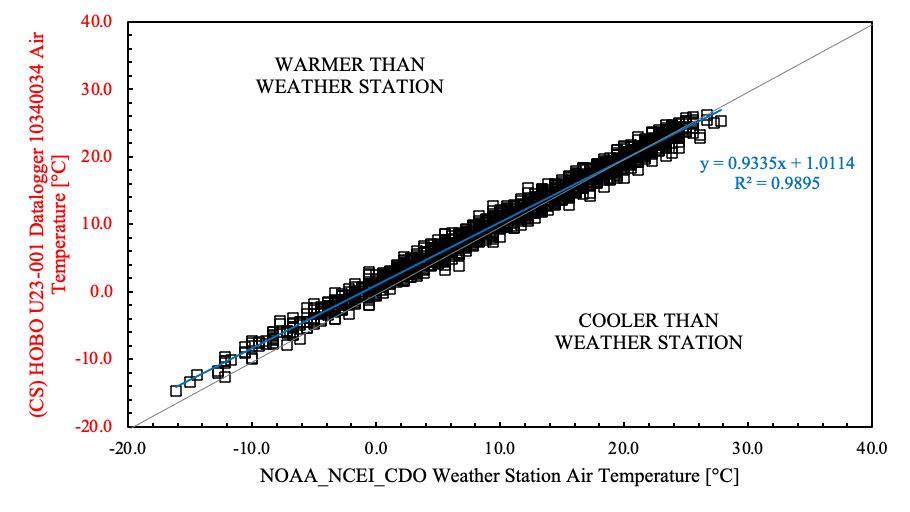

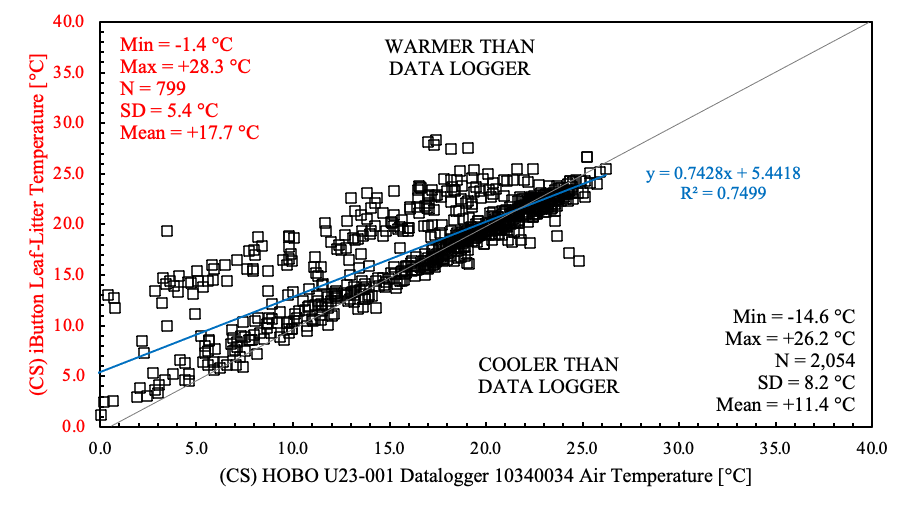


***Figure S1.*** Sources of near-surface air temperature data used to force the process-based model. (Top panel) Comparison between gridded and ground-based historical time series for a single year (2016). (Middle panel) Satellite-gauge gridded air temperature dataset versus NOAA NCEI CDO weather station air temperature data. (Bottom panel) Examples of linear transformations used to calculate daily air temperature under leaf litter.


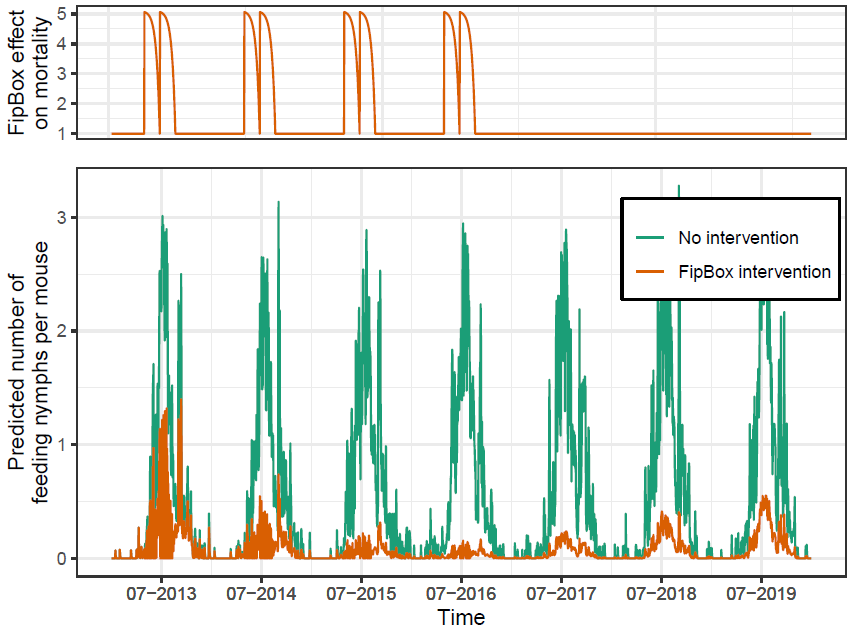


***Figure S2.*** Upper panel: Simulated increase (multiplicative factor depending on *f_F_* (equation 2)) in the daily per-capita mortality rate of feeding larvae (*μ_FL_*) and nymphs (*μ_FN_*), with the use of fipronil-based small rodent bait boxes (the FipBox intervention) during 4 consecutive years (2013 to 2016) in the model. Boxes are assumed to be implemented in early May each year and again 8 weeks later. Parameter *f_F_* was estimated using data from residential properties. Lower panel: Number of feeding nymphs per mouse predicted by the model between 2013 and 2019, in the “No intervention” scenario *versus* in the scenario with fipronil boxes implemented for 4 consecutive years (2013 to 2016).


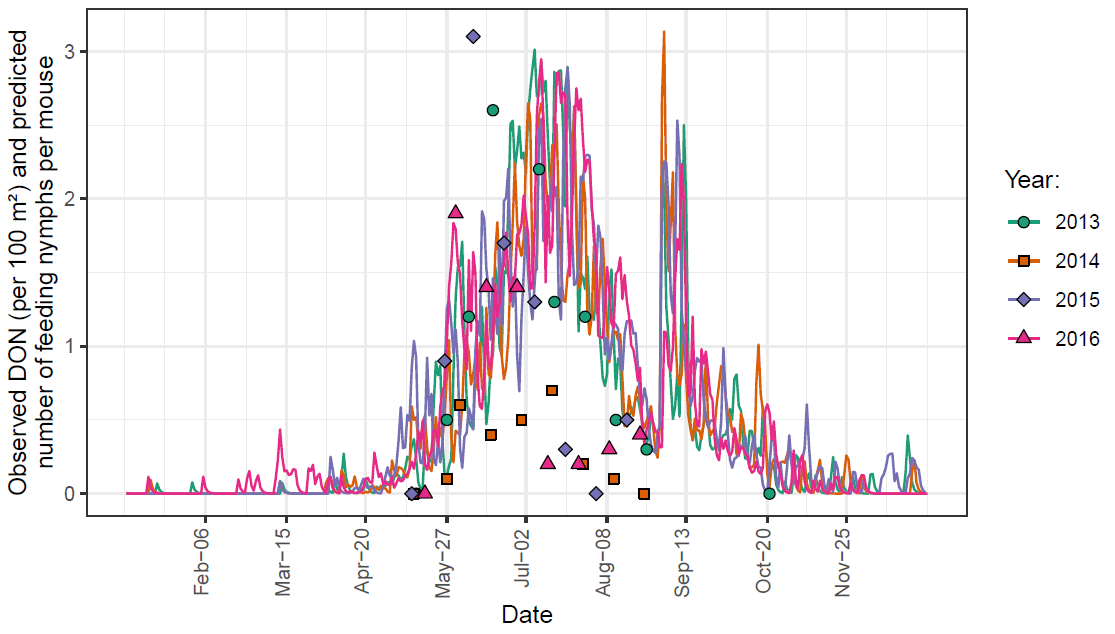


***Figure S3.*** 2013, 2014, 2015 and 2016 density of questing nymphs per 100 m^2^ (DON) in the properties that were used as experimental reference and received no intervention (dots) along with the number of feeding nymphs per mouse simulated by the dynamic tick population model for the same years (lines).


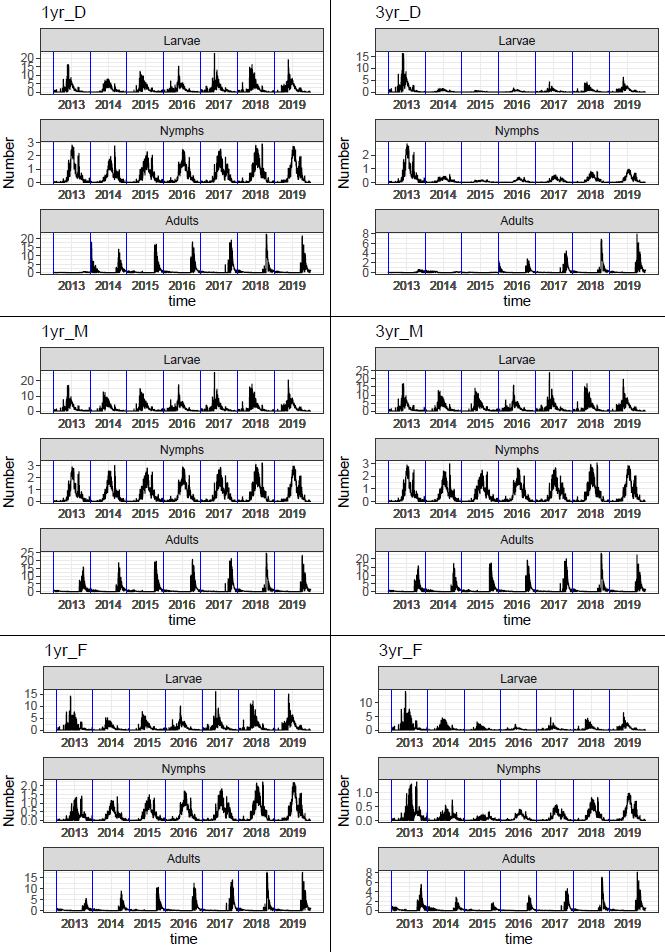


***Figure S4.*** Number of feeding larvae, nymphs and adults per host predicted by the model between 2013 and 2019, in different examples of scenarios: “1yr_D”, “1yr_M” and “1yr_F” depict the implementation of the “Deer Only”, “Met52 Only” and “FipBox Only” interventions, respectively, for one year in 2013. “3yr_D”, “3yr_M” and “3yr_F” depict their implementation for three years, between 2013 and 2015.


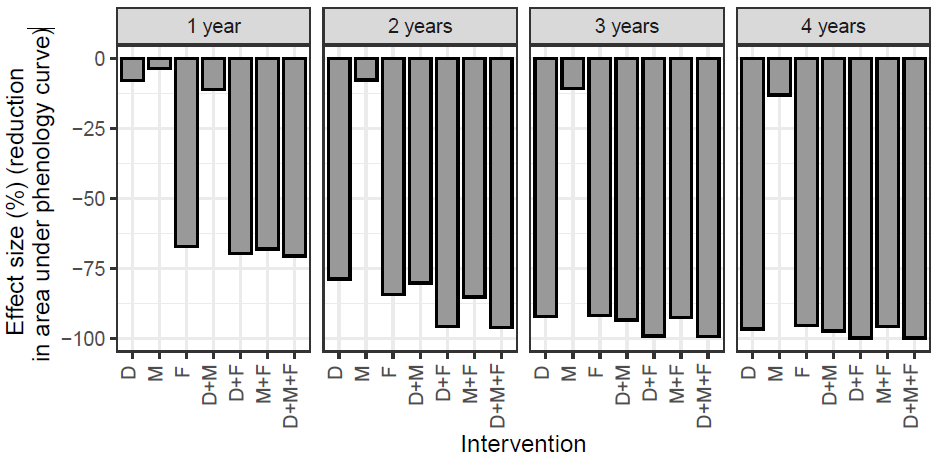


***Figure S5.*** Percent reduction of the area under the phenology curve (for feeding nymphs) in the last year of intervention, compared to the “no intervention” scenario. For each intervention (single and combined), different alternatives for the number of years of implementation (from one to four years) are presented. D: “Deer Only”; M: “Met52 Only”; F: “FipBox Only”; D+M: “Deer+Met52; D+F: “Deer+FipBox”; M+F: “Met52+FipBox”; D+M+F: “Deer+Met52+FipBox”.


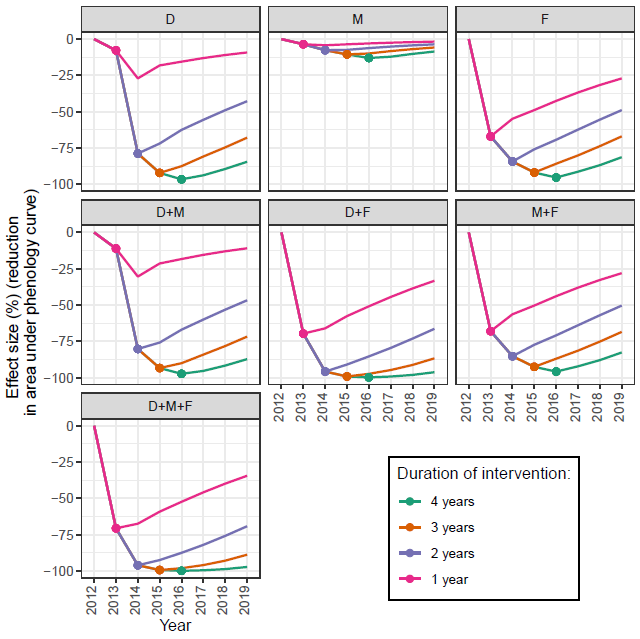


***Figure S6.*** Evolution of the percent reduction of the area under the phenology curve (for feeding nymphs) for each intervention, compared to the “no intervention” scenario. In our model simulations, interventions are all implemented in 2013, for one to four years. For each intervention and number of years, the dot represents the last year of intervention. D: “Deer Only”; M: “Met52 Only”; F: “FipBox Only”; D+M: “Deer+Met52; D+F: “Deer+FipBox”; M+F: “Met52+FipBox”; D+M+F: “Deer+Met52+FipBox”.


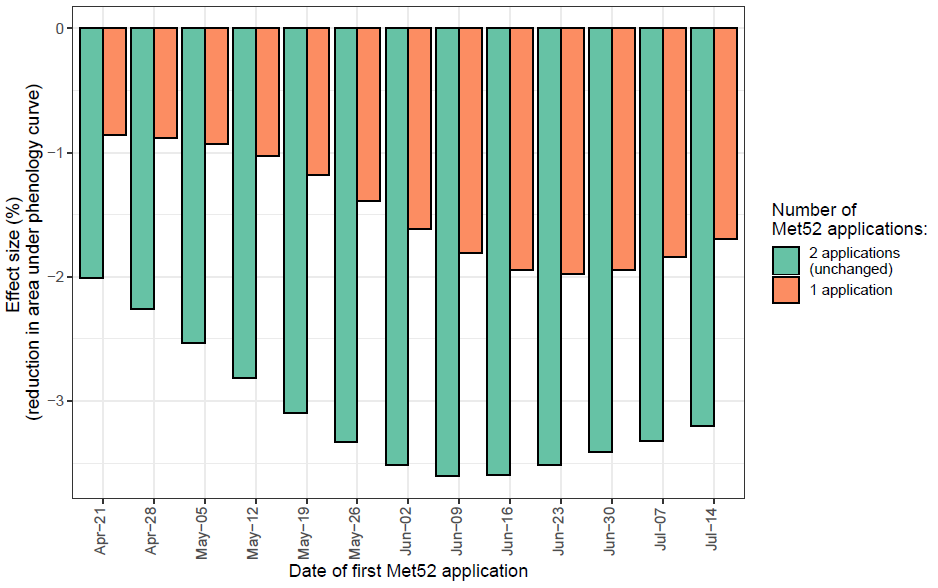


***Figure S7.*** Percent reduction of the area under the phenology curve (for feeding nymphs) in the only year of intervention, compared to the “no intervention” scenario, for different variations of the “Met52 Only” treatment. One or two Met52 applications are simulated, starting at different dates between April 21^st^ and July 14^th^. If done, the second Met52 application always takes place 29 days after the first one.
